# Outcomes of Bone Marrow Stimulation for Secondary Osteochondral Lesions of the Talus Equal Outcomes for Primary Lesions

**DOI:** 10.1177/19476035211025816

**Published:** 2021-06-24

**Authors:** Quinten G. H. Rikken, Jari Dahmen, Mikel L. Reilingh, Christiaan J. A. van Bergen, Sjoerd A. S. Stufkens, Gino M. M. J. Kerkhoffs

**Affiliations:** 1 Department of Orthopaedic Surgery, Amsterdam Movement Sciences, Amsterdam UMC—Location AMC, University of Amsterdam, Amsterdam, The Netherlands; 2 Academic Center for Evidence Based Sports Medicine, Amsterdam UMC, Amsterdam, The Netherlands; 3 Amsterdam Collaboration for Health and Safety in Sports, International Olympic Committee Research Center, Amsterdam UMC, Amsterdam, The Netherlands; 4 Department of Orthopedic Surgery, Albert Schweitzer Hospital, Dordrecht, The Netherlands; 5 Department of Orthopedic Surgery, Amphia Hospital, Breda, The Netherlands

**Keywords:** osteochondral lesion, OLT, bone marrow stimulation, microfracture, secondary treatment

## Abstract

**Objective:**

To compare clinical, sports, work, and radiological outcomes between primary and secondary osteochondral lesions of the talus (OLTs; <15 mm) treated with arthroscopic bone marrow stimulation (BMS).

**Design:**

Secondary OLTs were matched to primary OLTs in a 1:2 ratio to assess the primary outcome measure—the Numeric Rating Scale (NRS) during activities. Secondary outcomes included the pre- and 1-year postoperative NRS at rest, American Orthopaedic Foot and Ankle Society score, Foot and Ankle Outcome Score subscales, and the EQ-5D general health questionnaire. The rates and time to return to work and sports were collected. Radiological examinations were performed preoperatively and at final follow-up using computed tomography (CT).

**Results:**

After matching, 22 and 12 patients with small (<15 mm) OLTs were included in the primary and secondary groups, respectively. The NRS during activities was not different between primary cases (median: 2, interquartile range [IQR]: 1-4.5) and secondary cases (median: 3, IQR: 1-4), *P* = 0.5. Both groups showed a significant difference between all pre- and postoperative clinical outcome scores, but no significant difference between BMS groups postoperatively. The return to sport rate was 90% for primary cases and 83% for secondary cases (*P* = 0.6). All patients returned to work. Lesion filling on CT was complete (67% to 100%) in 59% of primary cases and 67% of secondary cases (*P* = 0.6).

**Conclusion:**

No differences in outcomes were observed between arthroscopic bone marrow stimulation in primary and secondary OLTs at 1-year follow-up. Repeat BMS may therefore be a viable treatment option for failed OLTs in the short term.

## Introduction

Arthroscopic bone marrow stimulation (BMS) is the most frequently performed operative treatment for primary osteochondral lesions of the talus (OLT).^1^ The aim of BMS is to reduce pain, improve clinical outcomes, and allow patients to resume physical activities and sports.^1,2^ Previous studies have reported that up to 82% of patients treated with primary BMS show successful clinical outcomes.^1,3^ Additionally, the return to preinjury level of sports has been found to be 79% following BMS.^4^ Arthroscopic BMS is exempt from the disadvantages of other more invasive secondary defect treatments, such as donor-site morbidity, the need for an osteotomy, and would still allow for additional surgical options if treatment fails.^5,6^

The use of arthroscopic BMS in secondary—that is, repeat BMS after failed primary surgery—OLTs is less frequent compared to primary cases.^1,3^ This can be attributed to the relatively inferior clinical results of secondary BMS reported in the literature.^3,7,8^ However, the number of studies with accompanying clinical evidence is limited and of low methodological quality, including a low number of patients. Additionally, no consensus exists on the effect of secondary BMS on sports outcomes, nor do studies directly compare clinical outcomes between primary and secondary BMS. This warrants further exploration of the efficacy of secondary BMS treatment on pain reduction, clinical outcomes, and the resumption of sports.

The primary objective of this study was to compare the 1-year postoperative Numeric Rating Scale (NRS) pain scores during activity between primary and secondary OLTs treated with arthroscopic BMS. It was hypothesized that no difference in postoperative NRS scores during activities would be observed between the 2 groups. The secondary aim was to compare the clinical, sports, work, and radiological outcomes between primary and secondary treatment groups.

## Methods

Approval by the local medical ethics committee at the Amsterdam UMC, location AMC, was obtained prior to the start of this study (Reference Number: MEC 08/326) and the study was performed in accordance with the Declaration of Helsinki. Patients were selected from a database constructed for a previously published randomized control trial (RCT), which was conducted between 2009 and 2014.^9^ The respective RCT investigated pulsed electromagnetic fields (PEMF) compared to placebo as adjuvant treatment for BMS, and included 36 patients in the PEMF group and 32 patients in the placebo group. The aforementioned study did not find statistically significant differences in clinical nor radiological outcomes between the PEMF and placebo groups at 1-year follow-up, and therefore both groups were merged into the database for the present study. The operative technique and postoperative rehabilitation protocol were previously described in detail.^9^

### Patient Selection

**Figure 1.**
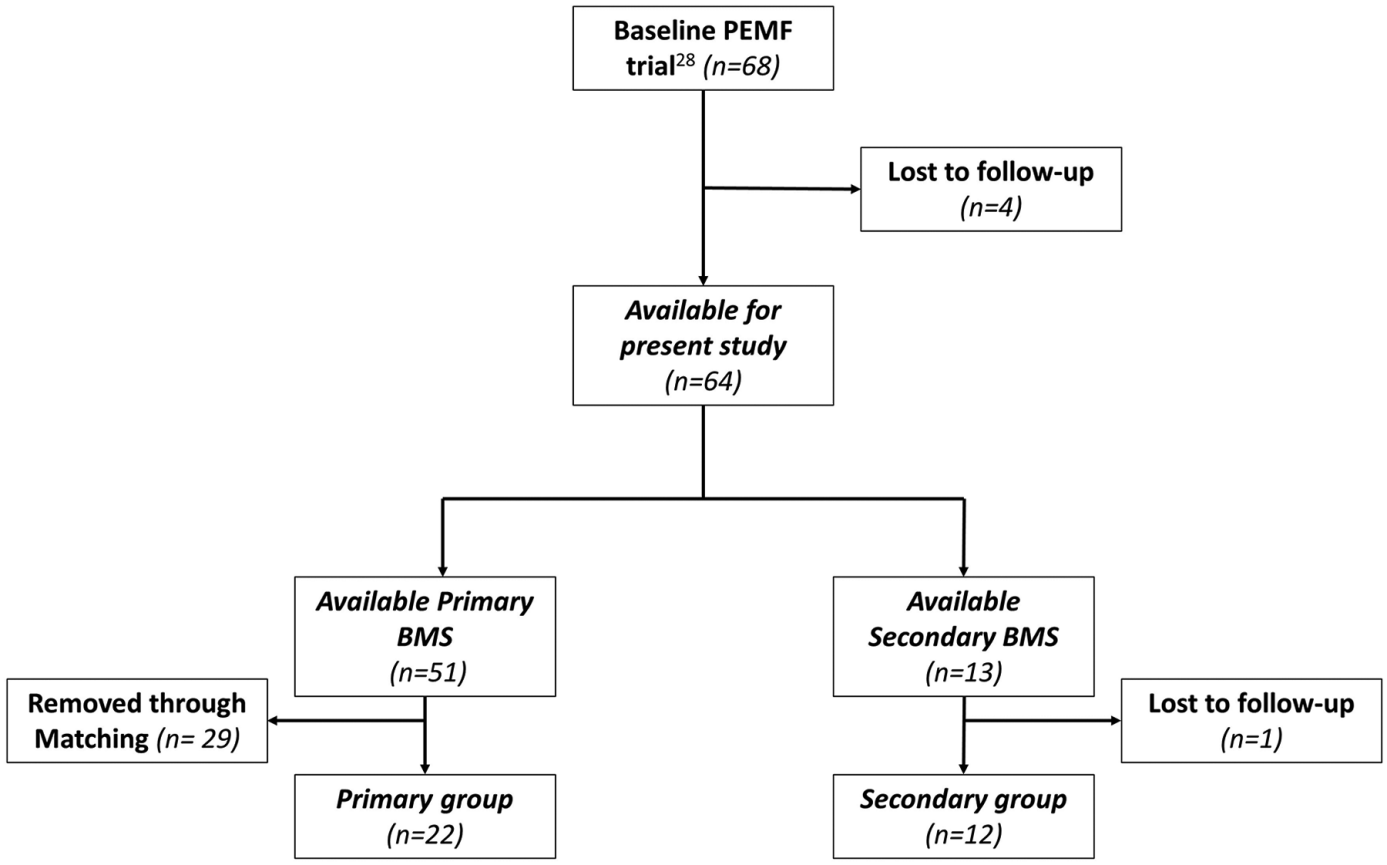
Flowchart of patient selection with inclusion and exclusion criteria.

Patients who underwent arthroscopic debridement and bone marrow stimulation (i.e., microfracturing) for either a primary or failed primary lesions (<15 mm in all dimensions as measured per computed tomography [CT] scan) were included (**Fig. 1**). The frequency of previous BMS procedures did not preclude inclusion. Exclusion criteria were set by the study from Reilingh *et al*.^9^ Patients who underwent repeat BMS (secondary group) were matched to patients who underwent primary BMS (primary group) as a control in a 1:2 ratio.^10^ Matching was based on the following prognostic variables: (1) lesion size as measured (in anterior-posterior, medial-lateral, and depth) per CT scan, (2) age, (3) body mass index (BMI), and (4) sex, as these factors have been shown to correlate with clinical outcomes following BMS.^11,12^

### Clinical Evaluation

#### Primary Outcome Measure

The primary outcome was defined as the difference of postoperative NRS^13^ during activities between the 2 groups. The NRS is a subjective pain scale from 0 (no pain) to 10 (worst pain imaginable).

#### Secondary Outcome Measures

Clinical outcomes were evaluated preoperatively and at 1-year follow-up. Secondary clinical outcomes concerned both pre- and postoperative comparisons in each respective treatment group, as well as a comparison between groups postoperatively, and included the NRS at rest, the American Foot and Ankle Outcome Society (AOFAS) score, the Foot and Ankle Outcome Score (FAOS), and the EQ-5D general health questionnaire. The AOFAS is a 100-point, physician administered, clinical outcome scale.^14^ Its subcategories consist of pain (40 points), function (50 points), and alignment (10 points). The FAOS is a patient-reported outcome measure consisting of 42 questions distributed over 5 subscales: symptoms, pain, activities of daily living, sport, and quality of life.^15,16^ The EQ-5D is a general health questionnaire, which reports the overall health of an individual on a 0% to 100% scale.^17^

### Sports and Work Evaluation

Preoperatively, the type of sport and athletic level (i.e., amateur, competitive, or professional) were recorded. Postoperatively, the evaluation consisted of the return to sports (RTS) rate in percentages and RTS time in weeks, type of sport, and level of activities. RTS was defined as the resumption of any sport at a minimum of presymptomatic level of sports, minus 1 point on the ankle Activity Score^18^ (AAS), maintained for a minimum of 30 days.^9^ Similarly, the pre- and postoperative occupation of patients and time to return to work were collected. Return to work was defined as resumption of work with normal activities without any deficits in work quality.^9^

### Radiological Evaluation

Radiological evaluation was performed by means of a CT scan preoperatively, at 2 weeks postoperatively, and 1 year postoperatively. A standardized imaging protocol concerned axial slices with 0.3 mm increment and 0.6 mm thickness, and multidirectional (coronal and sagittal) reconstructions of 1 mm.^9^ On preoperative imaging, lesions were graded according to the Berndt and Harty classification,^19^ and localization of the lesion was determined using a 9-grid scheme from Raikin and colleagues.^20^ Furthermore, lesion dimensions were measured in anterior-posterior, medial-lateral, and cranial-caudal (depth) directions, and the morphological aspects of the lesion were assessed (such as the presence of cysts). Subchondral bone plate characteristics (flushed or depressed) and the level of lesion filling (difference in lesion dimensions between 2 weeks postoperative and 1 year postoperative scans) were assessed on final follow-up imaging. Reilingh *et al*.^9^ previously established the intraobserver reliability for the radiological outcomes assessed to be excellent.

### Statistical Analysis

A sample size calculation for the primary outcome using a level of significance (α) of 0.05 and a 2-sided, 2-group Wilcoxon rank-sum test was performed with nQuery advisor 7.0 (Statistical Solutions Ltd., Boston, MA). The minimally clinical important difference (MCID) in the NRS for pain during activities of 2.0 (±1.3) between the primary and secondary groups with a power of 80% was chosen, as it correlates with a “much better” improvement in pain.^21-23^ Therefore, a minimum of 10 patients per group were needed.

Patient baseline characteristics were summarized using descriptive statistics with absolute numbers and percentages for categorical variables, and means with standard deviations for continuous variables. Data were assessed for normality using a Shapiro-Wilk test and inspected visually with histograms and box plots. Baseline characteristics and outcome variables were compared using a Fisher’s exact test for dichotomous variables and a chi-square test for ordinal variables. For continues outcomes, a Wilcoxon signed rank test was used for comparing pre- and postoperative outcomes per treatment group, and a Wilcoxon rank sum test for comparing pre- and postoperative outcomes between treatment groups. A univariate linear regression analysis was used to investigate the influence of covariates on clinical outcome scores. A 2-sided level of *P* < 0.05 was considered significant. Data analysis was performed using Stata 15 (StataCorp LP, College Station, TX).

## Results

### Patient Selection and Demographics

A total of 22 primary BMS patients and 12 secondary BMS patients were included for analysis after matching (**Fig. 1**). No significant differences in baseline patient and lesion characteristics between the primary and the secondary groups were present (**Table 1**).

**Table 1.** Patient and Lesion Characteristics at Baseline.

	Primary Group (*n* = 22)	Secondary Group (*n* = 12)	*P* Value
Sex, *n* (% male)	12 (56%)	8 (67%)	n.s.
Age (years), mean ± SD	30.5 ± 8.3	31.3 ± 7.5	n.s.
BMI (kg/m^2^), mean ± SD	24.1 ± 2.4	24.8 ± 2.6	n.s.
Smoking, *n* (%)	3 (14%)	4 (33%)	n.s.
Laterality, *n* (% right side)	7 (32%)	5 (42%)	n.s.
Previous ankle trauma, *n* (%)	19 (86%)	8 (67%)	n.s.
Previous ankle fracture, *n* (%)	3 (14%)	0 (0)	n.s.
Sports, *n* (%)	22 (100%)	12 (100%)	n.s.
Sports level, *n* (%)			n.s.
Professional	3 (14%)	1 (9%)	
Competitive	12 (54%)	7 (58%)	
Recreational	7 (32%)	4 (33%)	
Previous BMS procedures, mean (range)	—	1.4 (1-3)	n.a.
Time since previous BMS procedure (months), mean ± SD	—	31.9 ± 22.8	n.a.
Lesion characteristics			
Brendt and Harty, *n* (%)	Stage 2: 1 (5%)	Stage 1: 1 (8%)	n.s.
	Stage 3: 3 (13%)	Stage 2: 2 (17%)	
	Stage 4: 1 (5%)	Stage 5: 9 (75%)	
	Stage 5: 17 (77%)		
Presence of cyst, *n* (%)	11 (50%)	7 (58%)	n.s.
Size (mm), mean ± SD			
Anterior-posterior	11.1 ± 2.7	11.3 ± 2.6	n.s.
Medial-lateral	9.2 ± 2.5	9.1 ± 2.3	n.s.
Depth	7.0 ± 2.0	7.5 ± 1.4	n.s.
Location per zone^a^, *n* (%)			n.s.
Anteromedial (zone 1)	5 (23%)	1 (8%)	
Anterocentral (zone 2)	5 (23%)	2 (18%)	
Anterolateral (zone 3)	5 (23%)	3 (24%)	
Centeromedial (zone 4)	0	1 (8%)	
Centerocentral (zone 5)	3 (13%)	2 (18%)	
Centerolateral (zone 6)	4 (18%)	1 (8%)	
Posteriomedial (zone 7)	0	1 (8%)	
Posteriocentral (zone 8)	0	1 (8%)	
Posteriolateral (zone 9)	0	0	

n = number; SD = standard deviation; BMI = body mass index; BMS = bone marrow stimulation; n.s. = not significant; n.a. = not applicable.

a All zones not significant, zone distribution according to Raikin and colleagues.^20^

#### Primary Outcome

The median postoperative NRS during activities for the primary and secondary group was 2 (interquartile range [IQR]: 1-4.5) and 3 (IQR: 1-4), respectively, and did not show a significant difference (*P* = 0.46). Preoperatively, the NRS in rest (*P* = 0.09) and NRS during activities (*P* = 0.47) were not significantly different between both groups. Both treatment groups showed significantly lower pain scores during activity at final follow-up compared to the preoperative assessment (**Table 2**).

**Table 2.** Clinical Outcomes for the Primary and Secondary BMS Group.

	Primary Group (*n* = 22)			Secondary Group (*n* = 12)			Between Groups^a^
	Preoperative	1 Year Postoperatively	*P* Value	Preoperative	1 Year Postoperatively	*P* Value	*P* Value
NRS, median (IQR)							
Pain (activities)	8 (6 -10)	2 (1-4.5), *N* = *20*	<**0.01**	8.5 (8-10)	3 (1-4), *N* = *11*	**<0.01**	n.s.
Pain (rest)	2 (0-4)	0 (0)	**<0.01**	4 (2-4.5)	1 (0-2)	n.s.	n.s.
Satisfaction	—	7 (5-8)	n.a.	—	7 (6.5-8)	n.a.	n.s.
AOFAS, median (IQR)	72 (49-75)	90 (85-100)	**<0.01**	67 (46-69)	87 (79.5-100)	**<0.01**	n.s.
FAOS, median (IQR)							
Symptoms	58.9 (53.6-71.4)	75 (64.3-89.3)	n.s.	60.7 (50-71.4)	67.9 (48.2-82.1)	**0.03**	n.s.
Pain	63.9 (52.8-75)	91.6 (73.6-94.4)	**<0.01**	52.8 (45.8-66.7)	81.9 (63.9-91.7)	**0.01**	n.s.
ADL	69.1 (54.4-80.9)	95.6 (91.2-100)	**<0.01**	69.1 (47.8-87.5)	94.9 (72.8-98.5)	**0.02**	n.s.
Sport	42.5 (25-50)	80 (50-85)	**<0.01**	27.5 (20-52.5)	70 (42.5-75)	**0.01**	n.s.
QOL	34.4 (18.8-43.8)	53.1 (37.5-75)	**<0.01**	25 (18.8-28.1)	46.9 (28.1-68.8)	**0.01**	n.s.
EQ-5D, median (IQR)	78% (69.3-80.7)	84% (77.5-100)	**<0.01**	78% (29.8-77.5)	87% (79.3-100)	**<0.01**	n.s.
AAS, median (IQR)	5.5 (4-9)	7.5 (4-9)	n.s.	7.5 (4-9), *N* = 19	5 (4-8), *N* = *11*	n.s.	n.s.

NRS = Numeric Rating Scale; AOFAS = American Orthopaedic Foot and Ankle Society score; FAOS = Foot and Ankle Outcome Score; ADL = activities of daily living; QOL = quality of life; EQ-5D = EQ-5D general health questionnaire; AAS = Ankle Activity Scale; n.s. = not significant; n.a. = not applicable. Boldface: indicates a statistically significant difference between groups.

a Comparison of postoperative outcomes between primary and secondary groups.

#### Secondary Outcomes

##### Clinical outcomes

Preoperatively, no clinical outcome scores were significantly different between groups. Most secondary outcomes significantly improved in both groups at final follow-up in comparison to preoperatively, but did not show a significant difference between treatment groups at final follow-up (see **Table 2**). Patient age, sex, BMI, lesion size, the presence of cysts, or laterality did not significantly correlate with the primary and secondary clinical outcome scores.

At final follow-up, no major or minor complications were recorded. One patient from the secondary group was reoperated with a HemiCAP prosthesis for persistent pain complaints.

##### Resumption of sport and work

No statistically significant differences in sports outcomes were found between the 2 groups (**Table 3**). Patients returned to work at a median 5 weeks and 7 weeks for primary and secondary cases, respectively. Resumption of work was 100% in both groups.

**Table 3.** Sports and Work Resumption.

	Primary Group	Secondary Group	*P* Value
Return to sports rate, *n* (%)	20 (91%)	10 (83%)	0.6
Time to return to sports, median (IQR)	14 weeks (8-23)	19 weeks (13-26)	0.16
Return to work, *n* (%)	22 (100%)	12 (100%)	1.0
Time to return to work, median (IQR)	5 (2-6)	7 (5-11)	0.1

IQR = interquartile range.

##### Radiological outcomes

The baseline radiological lesion dimensions and characteristics are displayed in **Table 1**. At 1-year follow-up, CT scans were available for all patients except one patient from the secondary group. The subchondral bone plate status and filling were not significantly different (**Table 4**).

**Table 4.** CT Findings at 1-Year Follow-up.

	Primary Group	Secondary Group	*P* Value
Subchondral bone plate status, *n* (%)			0.6
Depressed	17 (77%)	10 (91%)	
Flushed	5 (23%)	1 (9%)	
Subchondral bone plate filling, *n* (%)			0.62
Complete (67% to 100%)	13 (59%)	8 (73%)	
Partially Complete (34% to 66%)	5 (23%)	1 (9%)	
Incomplete (0% to 33%)	4 (18%)	2 (18%)	

## Discussion

The main finding of this study is that no differences in clinical outcomes were observed between patients treated with primary and secondary bone marrow stimulation. Both treatment groups showed a significant and clinically relevant benefit from the intervention when compared to the preoperative situation. Moreover, similar return to sport and work rates were observed in both groups.

### Clinical Outcomes

On average, pain outcomes—in particular during activities—improved above the MCID threshold^23^ of 2 points in both treatment groups at 1-year follow-up. This threshold corresponds to a “much better” improvement in pain. Postoperative pain plays a major role in the limited success of repeat BMS, as reported by other authors.^24,25^ This finding is of clinical relevance as our findings do not fully coincide with the available literature, which shows poor results for patients treated with secondary BMS.^7,8,25^ However, when comparing the observed pain outcomes of the present study to the available literature one is constrained by the underreporting of the exact measure of pain. This study investigated pain scores during activities, while others did not report the circumstances of the perceived pain by study participants.^24-26^ It is therefore challenging to accurately compare these findings.

Clinical outcome scores, however, are universally reported, though limited.^3^ Yoon *et al*.^25^ found a mean Visual Analog Scale (VAS) for pain of 5.3 out 10 and a mean AOFAS score of 70 at 48 months follow-up in their study comparing repeat BMS to osteochondral autograft transplantation. In the aforementioned study, 32% of lesions treated with secondary BMS were larger than 150 mm^2^, which was in turn correlated with decreased clinical outcomes, a finding supported by the literature.^11,25^ However, the authors noted clinical outcomes—most notably the VAS pain score—for secondary BMS cases to decline over time and reported a clinical failure rate (defined as persistent pain or recurrent symptoms, repeat surgery, or AOFAS <80) of 53%. On the other hand, our findings concur with Savva *et al*.,^24^ who similarly concluded that secondary BMS yields good postoperative outcomes. Their retrospective case study found similar AOFAS scores for 12 patients at a mean follow-up of 6 years. In contrast, their study excluded cystic lesions as these have been associated with decreased clinical outcomes.^7,24^ In the present study 50% of primary lesions and 58% of secondary lesions included a cystic morphology, which did not significantly correlate with clinical outcomes.

From these findings an interesting question arises: Why do patients treated with secondary BMS not show successful outcomes after initial surgery? An explanation to the aforementioned question could be that during the initial procedure the lesion site was not amply debrided. During BMS it is key that full debridement of the lesion site and/or possible (subchondral) cysts takes place.^27^ Hereafter, adequate perforation of the sclerotic bone until bleeding needs to be achieved in order to facilitate sufficient bone filling and fibrocartilage formation.^27,28^ Another reason for BMS failure could be the inadequate healing of the subchondral bone plate, which has been seen as a crucial structure for cartilage regeneration.^29,30^ In the present study 77% and 91% of primary and secondary patients, respectively, were found to have a depressed subchondral bone plate at 1-year follow-up. This may lead to decreased fibrocartilage vitality over time and could lead to failure of the procedure at mid- to long-term follow-up. Preoperative and postoperative imaging, utilizing CT scans, is useful to determine lesion characteristics, arthroscopic access, and follow-up of the subchondral bone plate over time.^31,32^ Moreover, even though arthroscopic BMS is considered a simple procedure, surgeons should be mindful that lesion location (especially posteriorly located lesions) and morphology can impact the surgical access and level of difficulty of arthroscopic BMS procedures.

Possible augmentation with adjunct therapies such as autologous platelet-rich plasma (PRP) or bone marrow aspirate concentrate (BMAC) could further increase the clinical outcomes of BMS and might thereby increase the outcomes of repeat BMS cases.^33,34^ This could increase its indication in the future as small OLTs may not warrant more invasive surgical treatments.

### Return to Sports and Work

The return to sports rate for repeat BMS patients ranges from 38% to 67% but is rarely reported.^24,26^ This is in contrast to a systematic review by Steman *et al*.,^4^ which found 78% of patients treated with mostly primary BMS return to pre-injury level of sports, while 18% had some limitations in sporting activities. The present study found a higher RTS rate for both primary and secondary cases. When considering the return to sports time the present study observed no statistical difference, but did find a clinically relevant sooner return to sports for primary cases. A possible hypothesis for the longer RTS time could be the increased rehabilitation time after a more extensive repeat arthroscopy due to increased synovitis and scar tissue formation from previous arthroscopic ankle procedures. From the available literature it is not evidently clear what the impact of repeat BMS is on return to sports compared to a primary procedure.

All patients returned to work in this study. This is in accordance with the findings from Ogilvie-Harris and Sarrosa,^26^ who reported a similar return to work time for all patients.

### Radiological Outcomes

The subchondral bone plate has been identified to play an important role in osteochondral lesion healing as pain from an OLT arises from bony structures.^35^ In the present study, a high rate of subchondral bone plates was found to be depressed. This was also the case for a number of patients who reported high NRS pain scores (during activities) pre- and postoperatively, and could thus be considered failed cases. Inferior healing or an irregular bone plate morphology may increase the likelihood for the development of osteoarthritis.^36^ Additionally, deterioration of clinical outcomes over time because of the development of osteoarthritis—due to inferior wear characteristics of fibrocartilage—is a concern in the literature.^24,36,37^ Further research into the long-term effect of both primary and secondary BMS is needed to clearly establish the rate and prognostic factors for osteoarthritis after an OLT.

### Treatment Indication

Careful patient selection and education are critically important when considering secondary BMS for the treatment of OLTs as prognostic factors for successful outcome and long-term results have not yet been investigated. First, treatment indications for secondary BMS are similar to primary cases and are led by lesion and patient factors.^2^ Thus, lesions under 15 mm in diameter, noncystic lesions, and nonfixable lesions should preferably be treated with secondary BMS.^2^ Second, repeat BMS is a feasible option for patients with pain complaints and inability to work or participate in sports, who would benefit from surgical intervention but do not wish to undergo a more invasive procedure due to the risk of (long-term) complications and a relatively longer rehabilitation period. A personalized, evidence-based, approach is therefore needed when advising patients for the treatment of OLTs.^38^

### Methodological Considerations

The results of this study should be interpreted in the context of its design. The present study included a limited number of patients as matching was based on secondary BMS cases. However, matching primary and secondary BMS cases ensured no significant patient or lesion differences between groups were present in order to limit the effect of confounding covariates. Furthermore, the present study included a sufficient number of participants according to our power analysis. It must be stated, however, that this assumption cannot be made for the secondary outcome measures. Second, even though data were prospectively collected it was retrospectively analyzed. The results of the present study should therefore be interpreted carefully and in the context of the study design. Last, the present study had a follow-up of 1 year. As seen in previous literature, it may be that outcomes decrease over time.^25^ It is therefore of interest to further follow-up these patients.

### Clinical Relevance and Future Perspectives

The present study shows surgeons can consider repeat BMS for small OLTs and patients who do not wish to undergo a more invasive procedure. The treatment indication for failed primary OLTs may therefore increase in the context of individualized patient care. However, future research with larger sample sizes in a randomized controlled setting or prospective cohort is highly needed, as limited evidence for secondary BMS exists.^3^ The MCID in NRS can be used as a benchmark for further comparative research. Furthermore, studies assessing the effect of adjunct therapies and long-term follow-up outcomes are needed.

## Conclusion

No differences in postoperative pain scores during activities at 1-year follow-up for primary (median NRS: 2) and secondary OLTs (median NRS: 3) treated with arthroscopic bone marrow stimulation were observed. Similarly, no significant differences in secondary clinical, sport, work, and radiological outcomes were found between both groups. Repeat BMS may therefore be a viable treatment option for small (<15 mm), failed OLTs. The indication for secondary BMS should be considered carefully by patients and surgeons.
